# A pilot study demonstrating the identification of *Trypanosoma brucei gambiense* and *T*. *b*. *rhodesiense* in vectors using a multiplexed high-resolution melt qPCR

**DOI:** 10.1371/journal.pntd.0008308

**Published:** 2020-11-25

**Authors:** Gala Garrod, Emily R. Adams, Jessica K. Lingley, Isabel Saldanha, Stephen J. Torr, Lucas J. Cunningham

**Affiliations:** 1 Department of Vector Biology, Liverpool School of Tropical Medicine, Liverpool, United Kingdom; 2 Department of Tropical Disease Biology, Liverpool School of Tropical Medicine, Liverpool, United Kingdom; Makerere University, UGANDA

## Abstract

Human African Trypanosomiasis (HAT) is a potentially fatal parasitic infection caused by the trypanosome sub-species *Trypanosoma brucei gambiense* and *T*. *b*. *rhodesiense* transmitted by tsetse flies. Currently, global HAT case numbers are reaching less than 1 case per 10,000 people in many disease foci. As such, there is a need for simple screening tools and strategies to replace active screening of the human population which can be maintained post-elimination for Gambian HAT and long-term for Rhodesian HAT. Here, we describe the proof of principle application of a novel high-resolution melt assay for the xenomonitoring of *Trypanosoma brucei gambiense* and *T*. *b*. *rhodesiense* in tsetse. Both novel and previously described primers which target species-specific single copy genes were used as part of a multiplex qPCR. An additional primer set was included in the multiplex to determine if samples had sufficient genomic material for detecting genes present in low copy number. The assay was evaluated on 96 wild-caught tsetse previously identified to be positive for *T*. *brucei s*. *l*. of which two were known to be positive for *T*. *b*. *rhodesiense*. The assay was found to be highly specific with no cross-reactivity with non-target trypanosome species and the assay limit of detection was 10^4^ tryps/mL. The qPCR successfully identified three *T*. *b*. *rhodesiense* positive flies, in agreement with the reference species-specific PCRs. This assay provides an alternative to running multiple PCRs when screening for pathogenic sub-species of *T*. *brucei s*. *l*. and produces results in less than 2 hours, avoiding gel electrophoresis and subjective analysis. This method could provide a component of a simple and efficient method of screening large numbers of tsetse flies in known HAT foci or in areas at risk of recrudescence or threatened by the changing distribution of both forms of HAT.

## Author summary

With global cases of Human African Trypanosomiasis (HAT) reaching pre-elimination numbers, identifying areas of trypanosome transmission becomes increasingly important. With decreasing numbers of cases, the cost of identifying each positive case increases. Screening for trypanosomes in tsetse flies instead of in the human host allows for a faster, more cost-effective and efficient method of detecting areas in which parasite transmission is on-going. Molecular tools such as PCR play a critical role in molecular identification of parasites in vectors. However, at present, multiple PCRs are required to confirm the presence or absence of human infective trypanosomes. Here we present a novel assay which allows for the screening of both species of trypanosomes responsible for HAT in one assay. This method eliminates the need for multiple assays per sample, produces results which are easier to interpret, and reduces the risk of contamination of samples. We found the assay to be as sensitive as the current gold-standard PCR when evaluated on a subset of wild tsetse flies. With further field evaluation, this method could provide an alternative monitoring method for HAT, which can be used leading up to elimination and maintained once elimination has been achieved.

## Introduction

Human African Trypanosomiasis (HAT) is a potentially fatal disease caused by subspecies of *Trypanosoma brucei s*. *l*. transmitted by the bite of an infected tsetse fly (*Glossina* spp). HAT consists of two forms of the disease, each with its own distinct parasite, vectors, disease pathology, treatment and geographical distribution. Gambian HAT (gHAT), caused by *Trypanosoma brucei gambiense*, is a largely anthroponotic disease found across central and west Africa and accounts for the large majority of HAT cases (>97%) [1]. Gambian HAT can remain asymptomatic for months to years with symptoms often presenting once the infection has significantly advanced. Conversely, Rhodesian HAT (rHAT), caused by *Trypanosoma brucei rhodesiense*, is a zoonosis with occasional human infection, and represents less than 3% of all HAT cases. The World Health Organisation (WHO) has targeted the elimination of HAT as a public health problem by 2020, defined as less than 1 new case per 10, 000 inhabitants in at least 90% of endemic foci and fewer than 2000 cases reported globally [2]. Due to the zoonotic nature of rHAT, this WHO target is applicable to gHAT only. With gHAT on the brink of elimination and rHAT persisting in several foci across East and Southern Africa [3], it is crucial to identify any remaining active cases, foci of transmission and areas of resurgence. The epidemiology of the two forms of HAT differ greatly and therefore monitoring and screening strategies also differ. Monitoring gHAT is largely reliant on the screening of the at-risk human population and treatment of cases [4]. Accurate estimates of disease prevalence require high rates of coverage, which can be difficult to achieve, particularly in areas affected by conflict and political instability [5] and where prevalence approaches <1 case per 10,000. As a result, there has been emphasis on the development of rapid diagnostic tests (RDTs) and field-friendly screening tools [6–10]. In comparison to gHAT, there has been little progress or investment into the development of a screening tool for rHAT with current emphasis on passive case detection and control of the vector population. A reliance on passive detection results in a delay in the identification and treatment of infected individuals, both of which are crucial for the control of disease transmission.

With declining numbers of cases, active screening programmes are no longer cost-effective [11] and there is a need for a monitoring tool which can be maintained sustainably for rHAT and post-elimination for gHAT. Xenomonitoring, the screening of vectors for the presence of parasites, provides a potential alternative to host sampling. This method has already been successfully utilised as a surveillance tool within the Lymphatic Filariasis elimination programme [12–14]. Vectors are often routinely collected as part of vector control programmes and as such, making use of this material may prove to be more economical than active screening of either human or animal populations. Additionally, screening vectors for infection is often less time-consuming and with efficient processing, can provide a view of disease transmission in real-time. Microscopy has been traditionally used for vector screening due to its low cost, high specificity and ease of use in-field. However, the sensitivity of microscopy is variable, it is labour intensive and morphological identification of *T*. *brucei gambiense* and *T*. *b*. *rhodesiense* trypanosomes is not possible [15,16].

The development of molecular tools has provided alternatives to traditional screening methods. PCR is widely used for the detection of trypanosome DNA, with highly sensitive assays developed for *T*. *brucei s*. *l*. [17] which includes *T*. *b*. *gambiense* and *T*. *b*. *rhodesiense* along with the animal trypanosome *T*. *b*. *brucei*. Successful amplification indicates the presence of one of the members of *T*. *brucei* s. *l*. but does not identify which member of *T*. *brucei s*. *l*. is present. Identification of *T*. *b*. *gambiense* and *T*. *b*. *rhodesiense* is reliant on the detection of specific single-copy genes (TgsGP [18] and SRA [19,20] respectively) for each subspecies. For detection of these low copy genes, the presence of sufficient genetic material is crucial. A negative result may indicate the absence of the target species or simply that insufficient DNA is present. To differentiate between these two scenarios, primers have been designed to screen for other single-copy genes [21], namely the single-copy phospholipase-C (PLC) gene, present in all members of *T*. *brucei s*. *l*. [22–25]. Samples found to have sufficient DNA can then be screened using primers specific for *T*. *brucei s*. *l*. subspecies.

High-resolution melt analysis (HRM) is a post-qPCR analysis method which can be used to detect heterogeneity within nucleotide sequences and has previously been used for the detection of other parasite species [26–28]. A fluorescent dye is added to the PCR reaction which intercalates into double stranded DNA. Following amplification, the amplicon is heated gradually causing the strands to separate. Separation of the double strands releases the incorporated dye causing a drop in fluorescence. The rate of DNA strand disassociation and the temperature at which it separates (Tm) is dependent on the amplicon’s nucleotide sequence. Different sequences will have different melting temperatures which can then be used as a diagnostic identifier. HRM is a closed tube process resulting in a reduced risk of contamination and produces results in approximately two hours circumventing gel electrophoresis, making it a faster, more specific alternative to traditional PCR. Multiplexing allows for the screening of a number of targets simultaneously, making sample processing more efficient.

### Objectives

We aim to develop a qPCR HRM assay designed to distinguish between the two human infective sub-species of *T*. *brucei s*. *l*. To improve the reliability of the assay primers were included to determine if sufficient DNA is present for the sub-species primers to work. To meet this objective we first designed and identified suitable primers that are compatible with the HRM qPCR, followed by assay optimisation and evaluation of sensitivity and specificity. Finally, the assay was compared to the current gold standard PCR using field samples available from other studies (S1 Fig).

## Methods

### Ethics statement

Ethical approval for this work was obtained from the Commission for Science and Technology (Costech) in Tanzania (permit number 2016-33-NA-2014-233). The samples from Tanzania were collected under the auspices of a BBSRC funded project (“Life on the edge: tackling human African trypanosomiasis on the edge of wilderness areas”, BB/L019035/1; COSTECH research permit number 2014-280-NA-2014-223).

### Primers

In order to be compatible with a HRM qPCR assay, primers that produced an amplicon of 150–350 base pairs with distinct melt temperatures were sought. Based on the literature, novel *T*. *b*. *rhodesiense* primers were derived from the sequence of a sub-species-specific serum-resistance-associated (SRA) protein gene (accession number AF097331.1). The primers for *T*. *b*. *gambiense*, previously designed and published by Radwanska *et al*. [29] were found to be compatible with the HRM qPCR method. These primers target the sub-species-specific glycoprotein (TgsGP: accession number AJ277951). The third primer set was designed to identify the presence of sufficient genetic material. These primers amplify a single-copy phospholipase-C (PLC) gene found in all members of the Trypanozoon group [22–25].

### Assay optimisation

To optimise the new HRM assay, the specificity of the multiplexed assay was evaluated using DNA from a range of non-target trypanosome species: *Trypanosoma congolense* Savannah (Gam2), *T*. *congolense* Forest (ANR3), *T*. *congolense* Kilifi (WG84), *T*. *simiae* (TV008), *T*. *godfreyi* (Ken7), *T*. *vivax* (Y486) and *T*. *grayi* (ANR4). Additionally, cross-reactivity of *T*. *b*. *gambiense* and *T*. *b*. *rhodesiense* primers against non-target *T*. *brucei s*. *l*. sub-species (i. e. *T*. *b*. *rhodesiense*, *T*. *b*. *brucei* and *T*. *b*. *gambiense*) DNA was tested. Each species’ DNA was run in triplicate along with negative water controls and target species positive controls.

The analytical sensitivity of the assay was assessed using a tenfold dilution series of *T*. *b*. *gambiense* and *T*. *b*. *rhodesiense* DNA obtained from culture which ranged from an estimated 10^6^ to 10^1^ tryps/mL, with each concentration run in duplicate. Quantification of DNA in terms of trypanosomes was calculated using concentrations of DNA as read by a Qubit 2.0 fluorometer (Invitrogen) and an assumed approximate DNA quantity of 0.1pg per trypanosome [30]. TgsGP and SRA PCR were run alongside on the same dilution series for a direct comparison of sensitivities [21,29].

### Screening of field samples

A subsample of wild-caught tsetse were selected to validate the HRM performance on field samples. The flies had been captured in 2015–2016 in Tanzania as part of the study reported by Lord *et al* [31]. Full methods are described in Lord *et al* [31], but in brief, a total of 5,986 tsetse were trapped using odour-baited Nzi traps deployed at sites in Grumeti and Ikorongo wildlife reserve, and Serengeti National Park. Captured flies were stored individually in 100% ethanol at room temperature and brought to LSTM for analysis. DNA extraction was carried out using Genejet DNA purification kit (Thermo K0721) according to the manufacturer’s instructions. Flies were screened for *T*. *brucei s*. *l*. and *T*. *congolense* Savannah using TBR [17] and TCS PCR [32] respectively. Both primer sets target satellite repeat sequences specific to each species. Flies positive for *T*. *brucei s*. *l*. were then screened for *T*. *b*. *rhodesiense* using SRA PCR.

### PCR

A subsample of 96 flies positive by TBR PCR were used in this study, of which two had been previously found to be positive for *T*. *b*. *rhodesiense* [31]. The species composition of the tsetse was 69.8% *Glossina pallidipes* (67/96) and 30.2% *G*. *swynnertoni* (29/96). Of the 96 *T*. *brucei s*. *l*. positive flies, 11.5% (11/96) were also found to be positive for *T*. *congolense* Savannah. Flies were re-screened using TBR [17] and SRA PCR [21] to reconfirm the presence and integrity of parasite DNA. TBR PCR conditions were as follows: initial denaturation at 95°C for 10 minutes, 40 cycles of 95°C for 30 seconds, 63°C for 90 seconds and 72°C for 70 seconds followed by an extension step at 72°C for 10 minutes. SRA PCR conditions were: initial denaturation at 95°C for 10 minutes, 40 cycles of 94°C for 30 seconds, 63°C for 90 seconds and 72°C for 70 seconds followed by an extension step at 72°C for 10 minutes. Additionally, flies were screened for *T*. *b*. *gambiense* using TgsGP PCR [29] using the following conditions: denaturation at 95°C for 10 minutes, 40 cycles of 95°C for 30 seconds, 63°C for 90 seconds and 72°C for 70 seconds followed a 10 minute extension at 72°C.

### HRM qPCR

All 96 flies were screened using the novel multiplexed HRM qPCR. HRM reactions were run in a total volume of 12.5μl consisting of 2.5μl DNA template, 6.25 μl HRM Master Mix (Thermo-start ABgene, Rochester, New York, USA), 3.25 μl sterile DNase/RNase free water (Sigma, ST. Louis, USA) and 400nM of all forward and reverse primers were included in a multiplex. Reactions were carried out on a Rotor-Gene 6000 real-time PCR machine (Qiagen RGQ system). The following protocol was followed: denaturation at 95°C for 5 minutes followed by 40 cycles and denaturation for 10 seconds at 95°C per cycle, annealing and extension for 30 seconds at 58°C, and final extension for 30 seconds at 72°C. The melting step ran from 75°C to 90°C with a temperature increase of 0.1°C every 2 seconds. A threshold was set at 10% of the maximum normalized fluorescence (dF/dT) of the highest peak, with all peaks that both occurred at the diagnostic temperature (Tm) and crossed this threshold classified as positive.

## Results

### Primers

**Fig 1:**
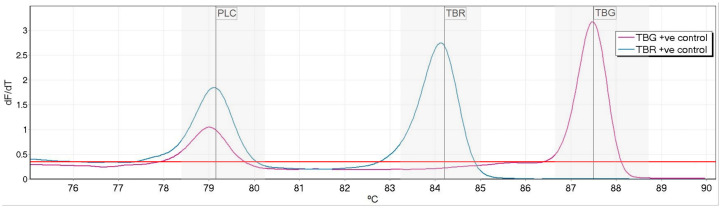
High resolution melt profile of phospholipase-C (PLC), *T*. *b*. *rhodesiense* (TBR) and *T*. *b*. *gambiense* (TBG) targets. Normalized fluorescence dF/dt is plotted against degrees °C (deg.) The melt temperatures for each peak were 79.1°C for PLC, 84.2°C for *T*. *b*. *rhodesiense and* 87.5°C for *T*. *b*. *gambiense*. The positivity threshold is shown in red and target specific bins indicated in grey.

One primer-pair per target was selected based on successful amplification, distinct Tm and production of a clear peak. Product sizes for each amplicon ranged from 134–319 base pairs (Table 1) with peak temperatures ranging from 79.2°C to 87.5°C. To allow for automated calling of peaks, bin widths of 1.5°C (0.75°C either side of diagnostic Tm) were set for each target (Fig 1).

**Table 1:** HRM and PCR primers used for identification of single-copy gene targets of *T*. *b*. *rhodesiense*, *T*. *b*. *gambiense* and *T*. *brucei s*. *l*. HRM primers were run in multiplex whilst PCR primers were run in singleplex. *This primer set was run in multiplex in HRM and in singleplex in PCR.

Primer	Species	Primer sequence 5’-3’	Product size (bp)	Product Tm (°C)	Reference	Assay
PLC1	Trypanozoon	CAGTGTTGCGCTTAAATCCA	319	79.1	This study	HRM
PLC2		CCCGCCAATACTGACATCTT				
TbRh1	*T*. *b*. *rhodesiense*	GAAGCGGAAGCAAGAATGAC	134	84.2	This study	HRM
TbRh2		GGCGCAAGACTTGTAAGAGC				
TgsGP1	*T*. *b*. *gambiense*	GCTGCTGTGTTCGGAGAGC	308	87.5	[29]	HRM and PCR*
TGsGP2		GCCATCGTGCTTGCCGCTC				
657	Trypanozoon	CGCTTTGTTGAGGAGCTGCAA GCA	324	-	[21]	PCR
658		TGCCACCGCAAAGTCGTTATT TCG				
SRA 02	*T*. *b*. *rhodesiense*	AGCCAAAACCAGTGGGCA	669	-	[21]	PCR
SRA 03		TAGCGCTGTCCTGTAGACGCT				

### Analytical specificity and sensitivity

No non-specific amplification was seen when the assay was challenged with a range of non-target trypanosome species, namely *T*. *congolense* (Savannah, Kilifi and Forest subgroups), *T*. *vivax*, *T*. *simiae*, *T*. *simiae* Tsavo, *T*. *godfreyi* and *T*. *grayi*. The limit of detection was found to be an estimated 10^4^ trypanosomes/mL for *T*. *b*. *gambiense* and *T*. *b*. *rhodesiense* using purified DNA from culture (S4 Fig). When the performance of the HRM was compared to TgsGP and SRA PCR on the same DNA dilution series, the HRM was as sensitive at detecting *T*. *b*. *gambiense* DNA and tenfold more sensitive for *T*. *b*. *rhodesiense* DNA (S2 and S3 Figs).

### Screening of field samples

**Fig 2:**
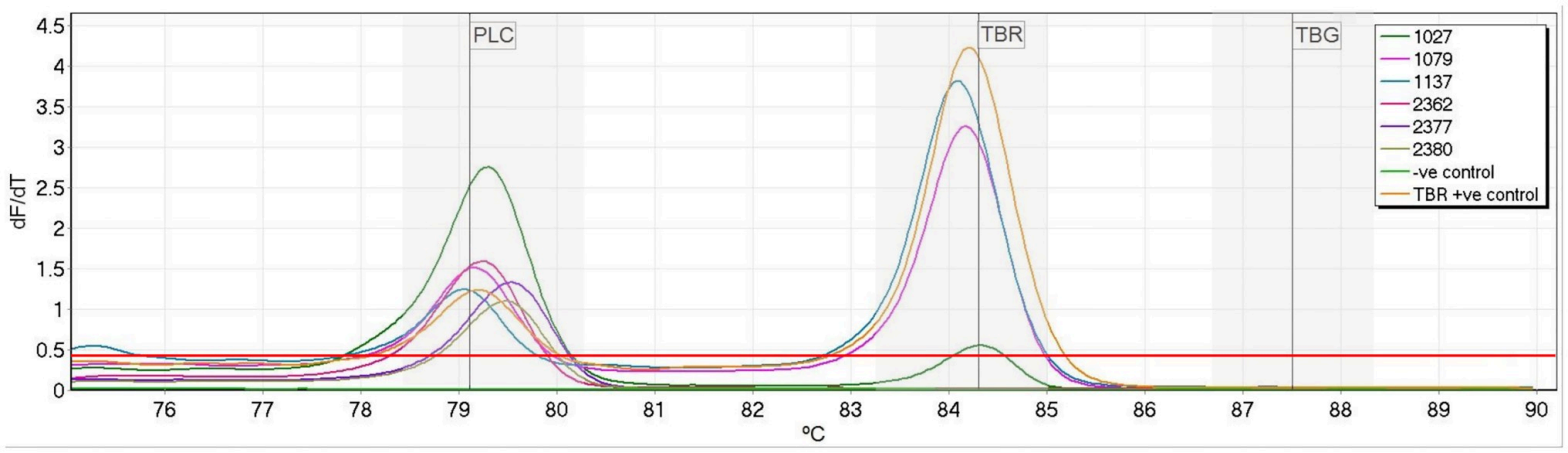
Melt profile of field samples. **Three samples were identified as *T*. *b*. *rhodesiense* due to the production of both PLC and TBR peaks (samples:1027, 1079 and 1137). Samples which only produced PLC peaks were considered to be *T*. *b*. *brucei* (samples: 2362,2377 and 2380). The TBR positive control was *T*. *b*. *rhodesiense* DNA and the negative control used was nuclease free water.** Normalized fluorescence dF/dt is plotted against degrees °C (deg.) with threshold indicated in red and species-specific bins shown in grey.

All flies (96/96) were positive by TBR PCR when initially rescreened. Using the multiplexed HRM, PLC positive control peaks were produced by 43 samples (44.8%), indicating sufficient target DNA for identification of either *T*. *b*. *gambiense* or *T*. *b*. *rhodesiense*. PLC PCR identified 19 samples (19.8%) with sufficient DNA quantity (Table 2). All samples which were positive by PLC PCR were also positive by HRM. None of the flies positive for *T*. *congolense* Savannah produced any non-specific amplification. Three flies (3.1%) were identified as positive for *T*. *b*. *rhodesiense* DNA both by HRM and PCR (Table 2), all of which also produced PLC peaks and were positive by PLC PCR (Fig 2). No flies were found to be positive for *T*. *b*. *gambiense* through either PCR or HRM. Flies that were positive for PLC but negative for *T*. *b*. *gambiense* or *T*. *b*. *rhodesiense* were considered to be infected with livestock trypanosome *T*. *b*. *brucei*.

**Table 2:** Breakdown of results of HRM and PCR on 96 field caught tsetse.

		HRM			PCR		
Tsetse species	Total screened (N)	PLC positive (%)	SRA positive (%)	TgsGP positive (%)	PLC positive (%)	SRA positive (%)	TgsGP positive
*G*. *pallidipes*	67	34 (50.7)	2 (3.0)	0	15 (22.4)	2 (3.0)	0
*G*. *swynnertoni*	29	9 (31.0)	1 (3.4)	0	4 (13.8)	1 (3.4)	0

## Discussion

Here we describe a novel high-resolution melt analysis for the detection and differentiation of *T*. *b*. *rhodesiense* and *T*. *b*. *gambiense*. This multiplexed assay screens for both pathogenic trypanosome species simultaneously with the addition of a third primer set to identify the presence of sufficient DNA to detect single-copy genes, acting as a positive control. The assay demonstrated high specificity with no cross-reaction with other non-target trypanosome species also transmitted by tsetse. The limit of detection of the HRM was lower than those reported in the literature for TgsGP [29] and SRA PCR [21]. However, when the three assays were compared directly against a dilution series of DNA, HRM was found to be as sensitive as TgsGP PCR and tenfold more sensitive than SRA PCR. The assay identified three wild caught tsetse flies to be positive for *T*. *b*. *rhodesiense* DNA. The three *T*. *b*. *rhodesiense* positives included two flies that had been previously identified by Lord *et al* [31]. All three flies including the additional fly that was identified by the HRM were positive by SRA PCR. However, the Ct value of the additional SRA positive was 30.5 in comparison to the two previously recorded positives which had Ct values of 21.1 and 21.8. This may indicate a lower DNA quantity and may represent a sample at the threshold of PCR detection. Of the 96 flies screened, 44.8% were positive for the universal-Trypanozoon PLC gene by HRM, indicating that they contained sufficient DNA for single-copy gene detection and therefore the detection of the SRA or TgsGP targets; if present. The remaining 55.2% of tsetse that were negative for the single-copy PLC gene can be assumed to have insufficient DNA present for the detection of the sub-species targets, we can therefore not rule these out as being negative for either *T*. *b*. *gambiense* or *T*. *b*. *rhodesiense*. Similar results have been reported in previous studies [33] and such a result is unsurprising when comparing the copy numbers, with TBR primers targeting a ~10,000 copy region and the sub-species primers targeting a single-copy gene, and presents an ongoing challenge. The two methods also differed in the number of flies positive for the single-copy PLC gene with the HRM method identifying a higher number of flies, indicating a greater sensitivity. This is likely due to the difficulty in identifying faint bands on a gel compared to the ability to better quantify a positive result using qPCR. When the Ct values of flies positive by HRM and PCR were compared to those that were only positive by HRM, the Cts of those positive by HRM only were slightly higher suggesting a potential failure of the PCR due to lower quantities of DNA in these samples (S5 Fig).

The method has three advantages over traditional PCR methods. First, the HRM time to result of ≤2 hours is faster than PCR followed by gel electrophoresis which can take over three hours for product amplification and visualisation. Second, this is a closed-tube assay which reduces contamination risk. Finally, it does not require interpretation of gel electrophoresis results. Through the use of detection bins, sample processing can also be automated, further speeding up and simplifying data analysis. The simple and fast nature of this method indicates it could be suitable for the high-throughput processing of tsetse. With prevalence of *T*. *b*. *gambiense* in tsetse from HAT foci predicted to be as low as 1 in 10^5^ [34], there is a need for a xenomonitoring tool which can be applied to large numbers of samples. At present, this method is not suitable to a low-resources setting because it requires a qPCR machine, however with further optimisation this technique could be applicable to more field-friendly technologies such as the Magnetic Induction Cycler (Mic) (Bio molecular Systems). This method has the potential to provide the basis of a real-time trypanosome transmission monitoring platform, enabling timely reactive measures by disease control programmes. Furthermore, with the risk of traditionally distinct geographical distributions of both Rhodesian and Gambian HAT changing due to the movement of livestock [35], human migration [36] and climate change [37–40], it may become increasingly important to screen simultaneously for both trypanosome species. The HRM allows for this and removes the risk of presumptive screening based on historic disease distributions.

### Study limitations

The authors acknowledge that the reliance of low copy genes for target identification is a limiting factor of the described diagnostic assay. However, at present these single copy genes are the only identifiers of members of the *T*. *brucei s*. *l*. and so pose a challenge to any diagnostic method based on these targets. This study was also challenged by a small number (96) of field caught flies from a rHAT focus and none from a gHAT focus where tsetse infected with *T*. *b*. *gambiense* might be present. Consequently, assay performance on field samples could only be evaluated for *T*. *b*. *rhodesiense*. Due to trypanosome prevalence in HAT foci predicted to be very low [34], obtaining sufficient field samples for any assay validation will be difficult. As control efforts such as active screening and treating the human population, combined with tsetse control, the numbers of infected tsetse will likely decline even further [41–44]. The authors are currently in planning to validate this method in a gHAT focus. Additionally, the field samples had only been screened for the presence of *T*. *brucei s*. *l*. and *T*. *congolense* Savannah. Studies of flies caught in this area suggest that these flies would have likely been infected with other trypanosome species [45,46] however this cannot be confirmed. Further validation using field caught flies with known infections with other non-target species would provide further evidence of the specificity of this method. Another limitation of this study was the comparison of only two methods for the identification of *T*. *b*. *rhodesiense* when there are alternative molecular methods available for HAT detection such as *Trypanozoon* sub-species specific LAMP [47,48] assays, which have demonstrated a high degree of sensitivity. Comparison between a wider range of methods would help assess HRM’s potential as a screening tool for human trypanosomes. A final limitation of this assay, as with all assays targeting DNA, is the inability to differentiate between a tsetse with an active infection or one with a transient one. Similarly, the assay is unable to determine whether a fly has a mature infection and is therefore capable of transmitting the disease. By using RNA as a target it may be possible to identify an active infection and determine what stage of infection it is; either mature or immature. Detailed analyses of RNA transcripts would be required to overcome this challenge.

In order to overcome these limitations follow-up studies will be conducted in order to more fully validate the HRM assay against larger field samples for both *T*. *b*. *gambiense* and *T*. *b*. *rhodesiense*.

In summary, we describe the development of a novel HRM assay for the detection and discrimination of human African trypanosomes in tsetse flies. The assay also incorporates an internal control, identifying samples with sufficient genomic material. The closed tube nature of the assay in addition to the relatively fast time and potential for automated calling lends itself to use in high throughput xenomonitoring surveillance campaigns for HAT.

## Supporting information

S1 Fig Molecular analysis workflow of field caught flies. (TIF)

S2 Fig Gel image showing the results of TgsGP PCR on a serial dilution of *T*. *b*. *gambiense* DNA from 10^6^ tryps/mL to 10^1^ tryps/mL. Marker (M) is 100bp (Invitrogen).
(TIF)

S3 Fig Gel image showing the results of SRA PCR on a dilution series of *T. b. rhodesiense* DNA on concentrations from 10^6^ tryps/ml to 10^1^ tryps/mL. Marker (M) is 100bp (Invitrogen).
(TIF)

S4 Fig Limit of detection of multiplexed HRM qPCR. DNA concentrations for *T*. *b*. *gambiense* and *T*. *b*. *rhodesiense* range from 10^6^ to 10^1^ tryps/mL. Positivity threshold is shown in red and target specific bins indicated in grey.
(TIF)

S5 Fig Ct values of flies positive by HRM only and both HRM qPCR and PCR. (TIF)
