# Supplementary figures and images for: A pilot study demonstrating the identification of Trypanosoma brucei gambiense and T. b. rhodesiense in vectors using a multiplexed high-resolution melt qPCR
Source: PLoS Negl Trop Dis. 2020 Nov 25;14(11):e0008308. doi: 10.1371/journal.pntd.0008308 (PMC7725321; doi:10.1371/journal.pntd.0008308)

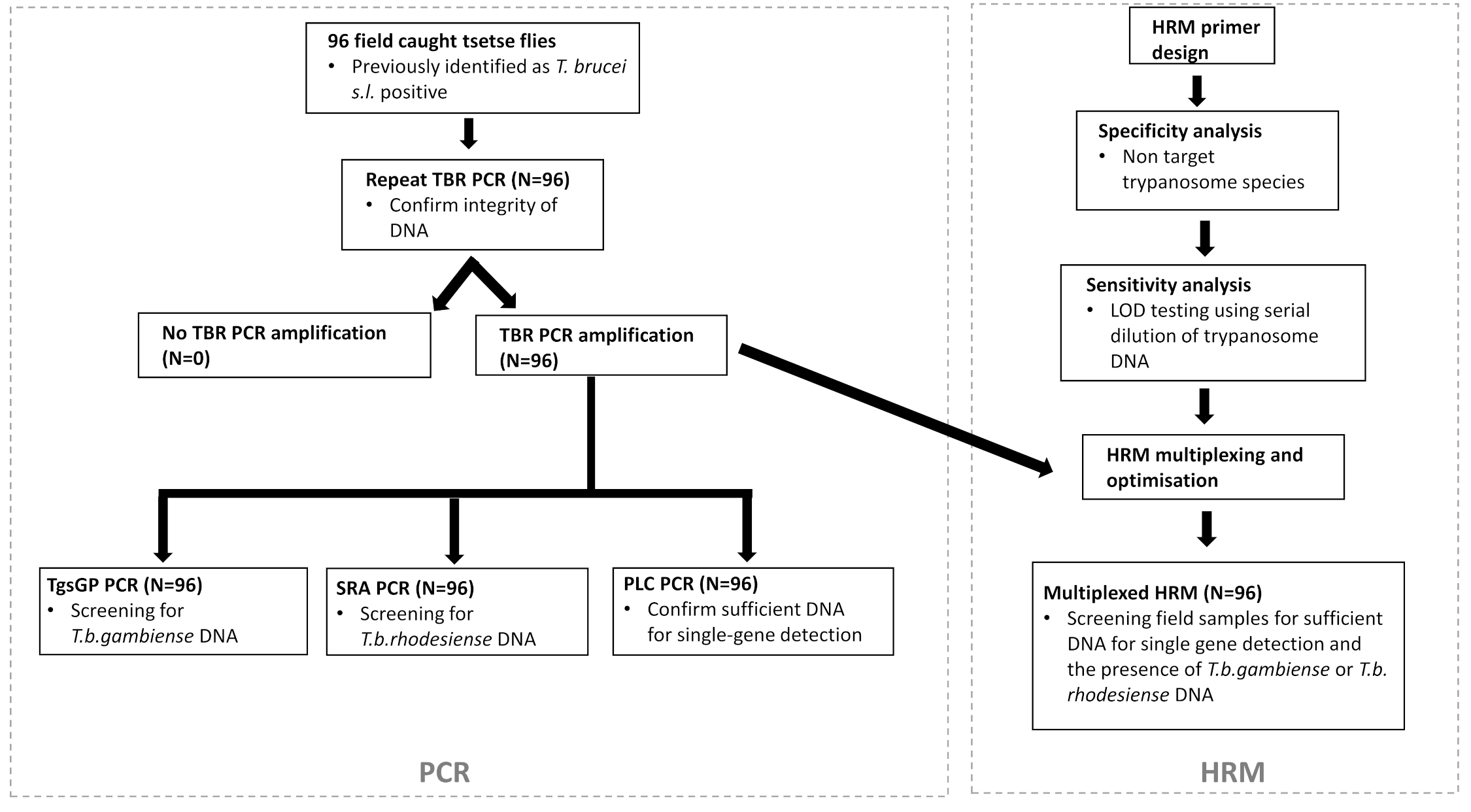

Supplement: S1 Fig — (TIF) [file pntd.0008308.s001.tif]

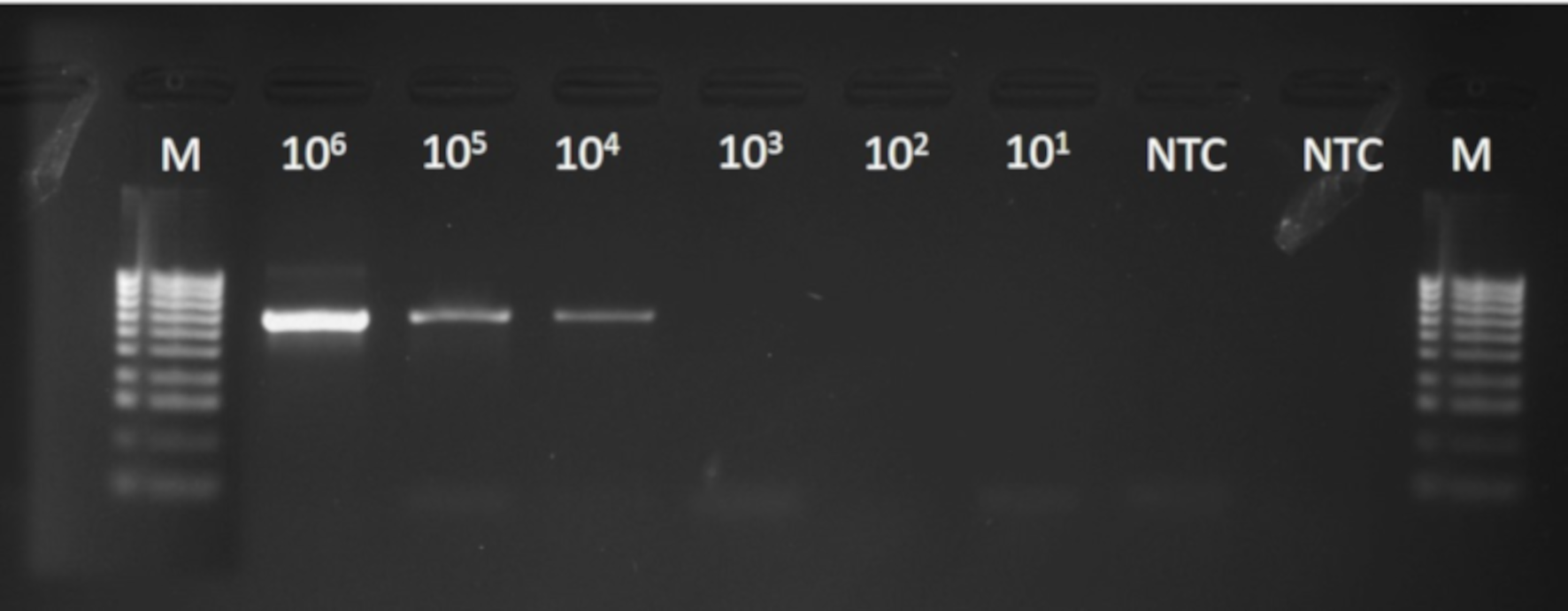

Supplement: S2 Fig — Marker (M) is 100bp (Invitrogen). (TIF) [file pntd.0008308.s002.tif]

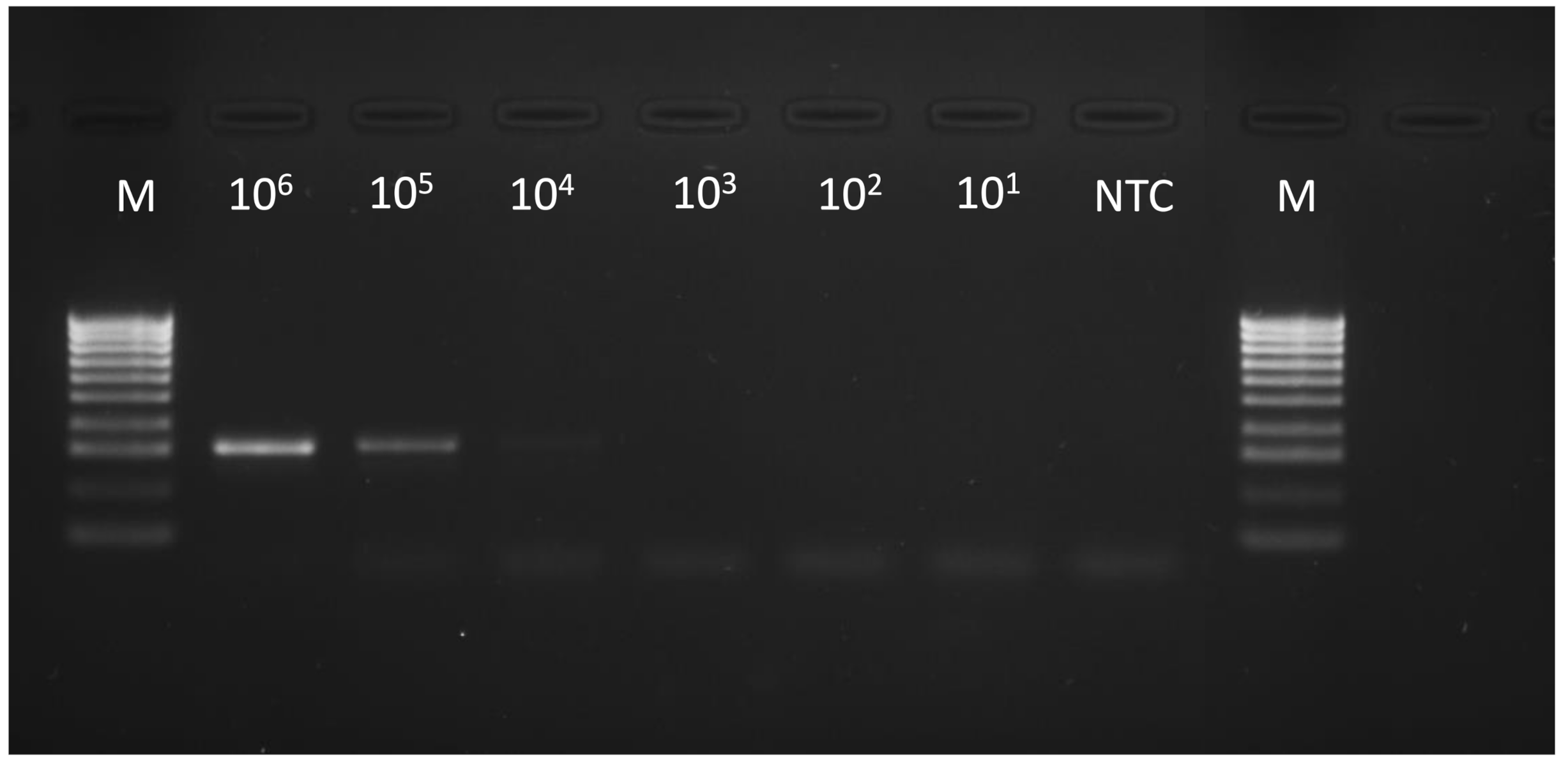

Supplement: S3 Fig — Marker (M) is 100bp (Invitrogen). (TIF) [file pntd.0008308.s003.tif]

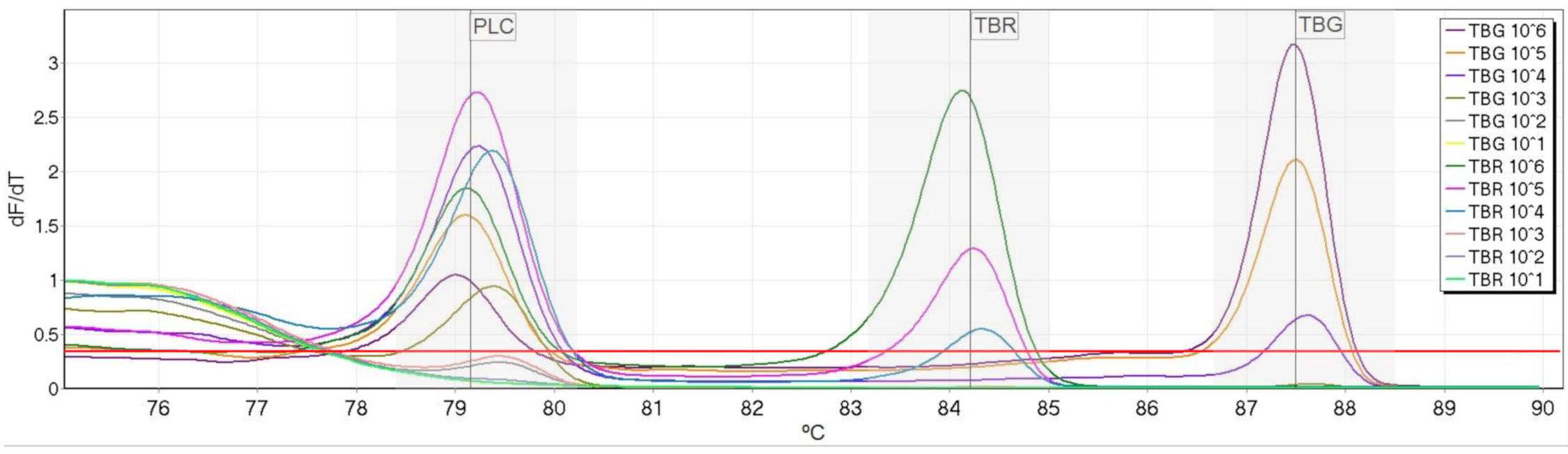

Supplement: S4 Fig — Positivity threshold is shown in red and target specific bins indicated in grey. (TIF) [file pntd.0008308.s004.tif]

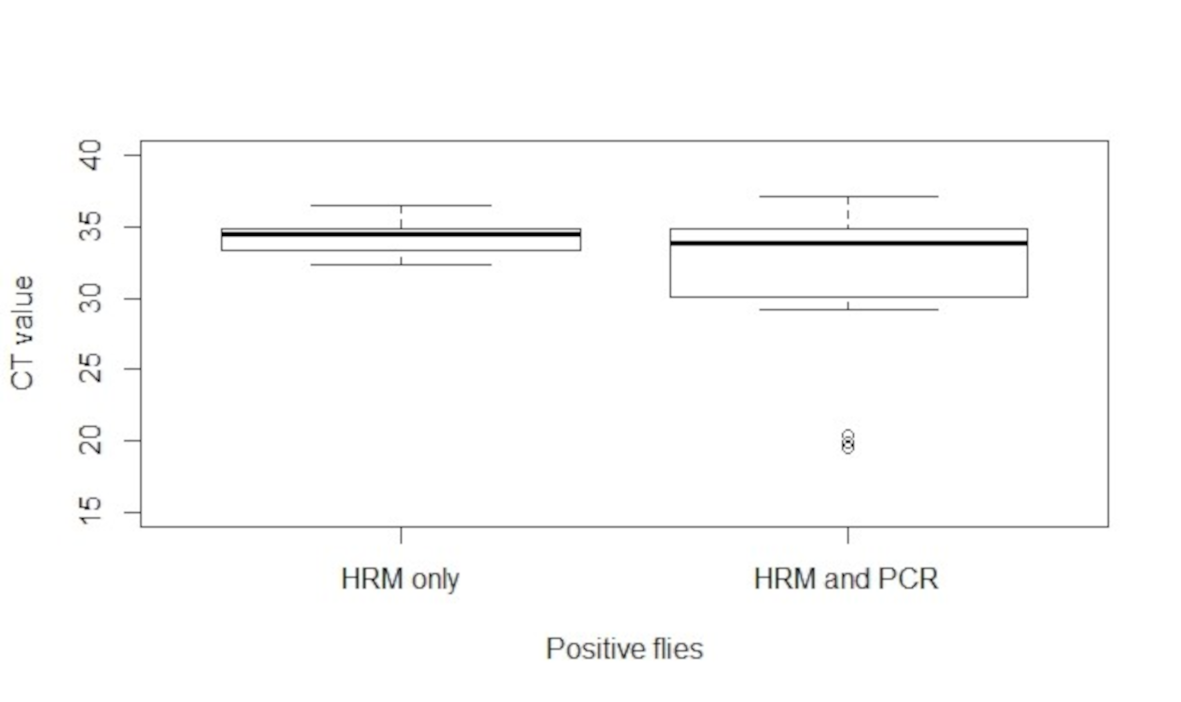

Supplement: S5 Fig — (TIF) [file pntd.0008308.s005.tif]
